# BgDB: a comprehensive genomic resource information system of bitter gourd for accelerated breeding programme

**DOI:** 10.1093/database/baaf039

**Published:** 2025-09-24

**Authors:** Princy Saini, Ankita Singh, Tilak Chandra, Dheeraj Kumar Chaurasia, Kunal Chaudhary, Priyanka Jain, G Boopalakrishnan, Sarika Jaiswal, Shyam Sunder Dey, Tusar Kanti Behera, Ulavappa Basavanneppa Angadi, Mir Asif Iquebal, Dinesh Kumar

**Affiliations:** Division of Agricultural Bioinformatics, ICAR—Indian Agricultural Statistics Research Institute, Library Avenue, PUSA, New Delhi 110012, India; Division of Agricultural Bioinformatics, ICAR—Indian Agricultural Statistics Research Institute, Library Avenue, PUSA, New Delhi 110012, India; Technology Innovation Institute, P.O.Box: 9639, Yas Island, Abu Dhabi, United Arab Emirates; Division of Agricultural Bioinformatics, ICAR—Indian Agricultural Statistics Research Institute, Library Avenue, PUSA, New Delhi 110012, India; Indian Institute of Technology Delhi, Hauz Khas, New Delhi, Delhi 110016, India; Division of Agricultural Bioinformatics, ICAR—Indian Agricultural Statistics Research Institute, Library Avenue, PUSA, New Delhi 110012, India; Division of Agricultural Bioinformatics, ICAR—Indian Agricultural Statistics Research Institute, Library Avenue, PUSA, New Delhi 110012, India; Division of Vegetable Science, ICAR—Indian Agricultural Research Institute, Pusa Campus, New Delhi 110012, India; Division of Agricultural Bioinformatics, ICAR—Indian Agricultural Statistics Research Institute, Library Avenue, PUSA, New Delhi 110012, India; Division of Vegetable Science, ICAR—Indian Agricultural Research Institute, Pusa Campus, New Delhi 110012, India; ICAR—Indian Institute of Horticultural Research, Hessaraghatta Lake Post, Bengaluru 560 089, India; Division of Agricultural Bioinformatics, ICAR—Indian Agricultural Statistics Research Institute, Library Avenue, PUSA, New Delhi 110012, India; Division of Agricultural Bioinformatics, ICAR—Indian Agricultural Statistics Research Institute, Library Avenue, PUSA, New Delhi 110012, India; Division of Agricultural Bioinformatics, ICAR—Indian Agricultural Statistics Research Institute, Library Avenue, PUSA, New Delhi 110012, India

## Abstract

Bitter gourd, scientifically known as *Momordica charantia* L. with 2*n* = 22, is a widely recognized medicinal vegetable, renowned for its multifaceted health benefits, primarily acclaimed for its lipid- and glucose-lowering effects. Its growing demands as a food source and for industrial applications necessitate value addition in ongoing breeding initiatives to enhance genotypic traits in multifarious ways. A thorough understanding of the underlying molecular footprint is warranted for characterization, which still remains underexplored relative to other cash crops. Though a chromosome-level genome assembly of bitter gourd is available, scattered and fragmented information becomes an obstacle for assisted breeding and gene editing. Therefore, it is crucial to further dissect structural and molecular variants, noncoding RNAs (ncRNAs), transcription factors, and transcripts from whole-genome and resequencing projects. The present study leads to the development of a comprehensive genomic resource, BgDB (Bitter Gourd Resource Database) at a single platform, vital for advanced bitter gourd breeding programmes for raising bitter gourd varieties with traits of significant social and economic value. BgDB, available at https://bgdb.daasbioinfromaticsteam.in/index.php, is a user-friendly, three-tier database that offers a comprehensive interface with detailed analysed information, including 114 598 transcripts, 4914 differentially expressed genes, 32 570 predicted simple sequence repeat markers, and 162 850 primers for downstream applications. It also catalogues extensive annotations of bitter gourd-specific single nucleotide polymorphisms/insertions and deletions, long noncoding RNAs, circular RNAs, microRNAs, 1220 transcription factors, 295 transcription regulators, and 146 quantitative trait loci (QTL) distributed throughout the chromosomes. This genomic resource is poised to significantly advance genetic diversity analyses, population and varietal differentiation, and trait optimization. It further facilitates the exploration of regulatory ncRNA elements, key transcripts, and essential transcription factors and regulators. The discovery of QTL will aid in the development of improved bitter gourd varieties in the endeavour of enhanced productivity. Beyond comprehensive datasets, the future integration of multi-omics resources could profoundly advance and fully unlock the potential of databases.

**Database URL**: https://bgdb.daasbioinfromaticsteam.in/index.php

## Introduction

Bitter gourd (*Momordica charantia* L.) is a tropical and subtropical vine recognized for its multifaceted nutritional potential owing to inherent reservoirs of various phytochemical and bioactive compounds [1]. Although primarily cultivated for its edible fruit, various parts of the plant also offer substantial functional advantages [2, 3]. The plant’s extracts exhibit a broad spectrum of bioactivities, including anti-inflammatory, antiviral, antibacterial, antihepatotoxic, antidiabetic, anticancer, hypocholesterolemic, antidementia, and antiulcerogenic effects. These properties are attributed to its rich phytochemical composition, which encompasses phenols, flavonoids, isoflavonoids, terpenes, anthraquinones, and glycosides. Extensive research has explored to establish the bioactivity of these compounds both *in vitro* and *in vivo* [1, 4–6]. Its significant role in traditional medicine and modern health practices is well documented, with a historical background of use in various cultures. It is particularly renowned as ‘vegetable insulin’ due to its notable hypoglycaemic effects [5, 7]. Recent developments highlight its potential for industrial applications, including the sustainable use of its residues in cotton dyeing [8], extraction of seed oils rich in bioactive compounds for pharmaceutical and nutraceutical purposes [2], and applications in industrial enzyme production for pollution control [9, 10]. Despite its often underrated status due to its distinctively bitter taste, which can limit consumer preference, bitter gourd’s unique flavour is increasingly recognized as a desirable attribute. As knowledge of its benefits grows and industrial applications expand, the acceptance and utility of bitter gourd are likely to increase significantly. Its cultivation is spreading from tropical and subtropical regions to a more global scale, making it a notable contributor to agricultural economies worldwide.

Due to its inherent nutritional benefits and medicinal properties, bitter gourd is a valuable addition to a balanced diet. However, its production struggles to keep pace with growing demand, often resulting in increased prices and strain in off-season production. This production challenge is compounded by the crop’s specific growing requirements and its susceptibility to various environmental and biological stressors, including climate conditions, soil types, water needs, and pest and disease pressures. Additionally, the genetic complexity and limited diversity of bitter gourd pose significant obstacles to conventional breeding efforts. Recent advancements in molecular breeding and innovative technologies, such as CRISPR–Cas9 (clustered regularly interspaced short palindromic repeats–CRISPR-associated protein 9), offer potential solutions to these challenges and could enhance breeding outcomes for bitter gourd. To achieve these improvements, it is essential to understand the underlying molecular mechanisms and genetic footprints. The availability of a chromosome-based genome assembly of bitter gourd has enabled the extraction of novel genomic information [11]. Despite these advancements, a significant gap persists in the integration and utilization of available genomic data. Previous studies, such as candidate gene-based simple sequence repeat (SSR) and single nucleotide polymorphism (SNP) markers for gynoecy in bitter gourd (*M. charantia* L.) [12], have contributed valuable insights into trait-specific molecular markers. However, there remains a lack of a unified platform that brings together diverse genomic information such as genome-wide variants, regulatory sequences, transcription factors, and other genetic elements in a manner that is accessible and practical for breeding and biotechnological applications. This fragmentation makes it challenging for researchers and breeders to fully leverage genetic knowledge, thereby slowing the development of improved bitter gourd varieties with desirable traits. Therefore, it is crucial to conduct further functional dissection of molecular footprints derived from whole-genome and resequencing projects, which can then be made available to breeders and biotechnologists. To address this gap, it is necessary to develop a comprehensive, robust, multipurpose resource system that integrates dissected molecular footprints from the latest omics datasets, including whole-genome variants, mature noncoding RNAs (ncRNAs), transcription factors, and other regulatory networks. In response, we have initiated the development of a comprehensive genomic resource database designed for the scientific community, with a focus on addressing previously unexplored objectives in bitter gourd research. Such resources are vital for advancing breeding programmes and facilitating the development of high-quality bitter gourd varieties with traits of significant social and economic value. Additionally, the bitter gourd genomic resource provides an intuitive platform for accessing exhaustive genomic data available, facilitating future research and breeding initiatives.

## Materials and methods

### Raw data curation, quality check, and processing

**Figure 1.**
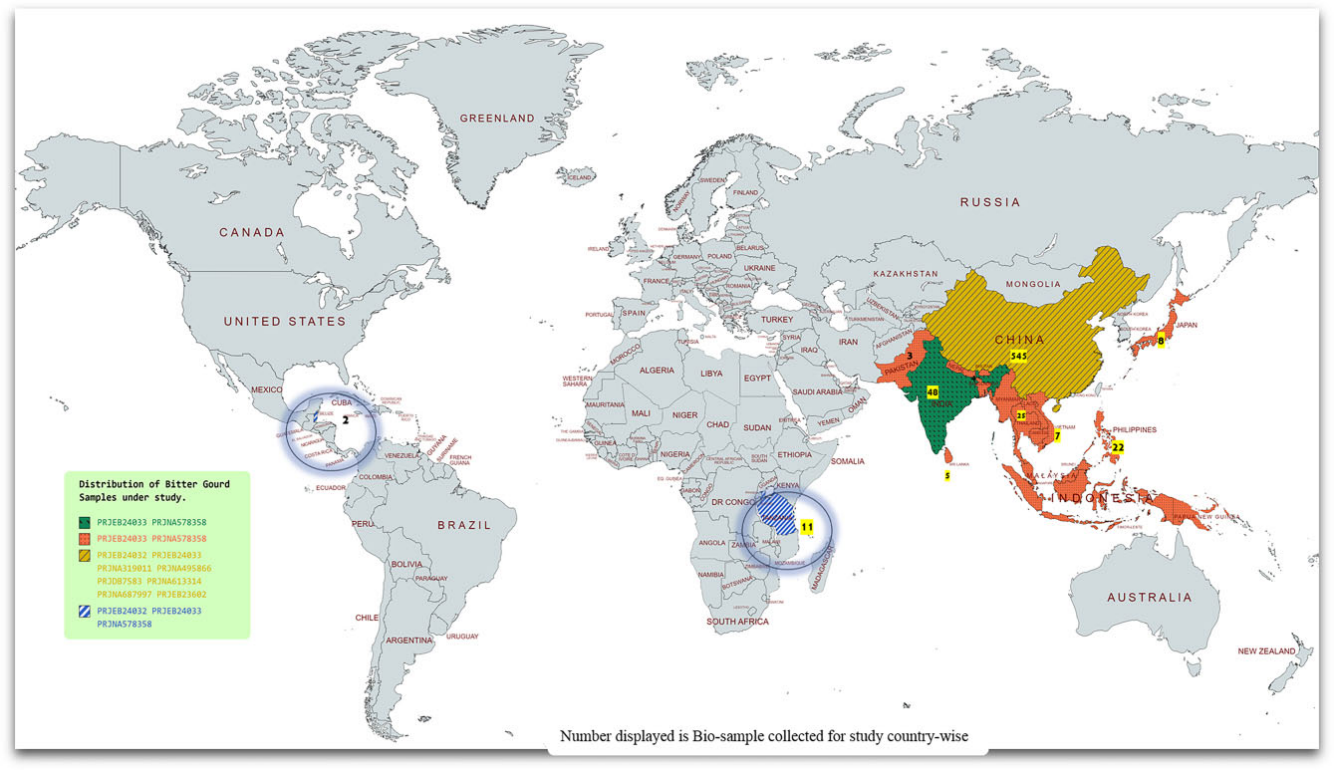
A country-wise representation and distribution of bitter gourd assemblies and biological samples collected for the study. The biological samples for this study were sourced from both the countries of origin and semidomesticated regions, spanning across Asia, Africa, and Mexico. This geographical distribution was selected to explore the evolutionary significance of bitter gourd, a notable medicinal vegetable, and to enhance understanding of its genetic diversity and adaptation across different environmental contexts.

The three published genome assemblies of bitter gourd, namely GCA_013281855.1, GCA_001995035.1, and GCA_900491585.1, encompassing 792 genotyping-by-sequencing (GBS) and 35 RNA-seq datasets, were used. Mcharantia_2.0 (2020), selected as the primary reference for its improved scaffold contiguity and annotation quality, and the most recent chromosome-based genome sequence assembly were obtained from NCBI (https://www.ncbi.nlm.nih.gov/) [11] (Fig. 1). The GBS libraries from 792 genotypes were assessed for quality using FastQC [13] with Phred score ≥30 and GC content >40%. *De novo* assembly of each library was conducted using SPAdes version 3.13.0 [14] with default settings. Subsequently, Trimmomatic version 0.39 [15] was employed to remove potential contaminants, such as adaptor sequences and low-quality reads with Phred scores below 30. The resulting clean reads were then prepared for mapping and further downstream analysis.

### Variant analysis and primer design

High-quality clean reads were aligned to the reference genome assembly of bitter gourd (Mcharantia_2.0) using Burrows–Wheeler Aligner (BWA) [16]. The aligned files were sorted, and polymerase chain reaction (PCR) duplicates were removed. Variant calling was performed using the standard bcftools pipeline. High-quality variants [SNPs and Indels (insertions and deletions)] were identified by applying stringent filtering criteria: a minimum quality score of 30, a minimum depth of 10, and a maximum missing rate of 0.9. These high-quality variants were then mapped to the 11 chromosomes of bitter gourd. SSRs from the Mcharantia_2.0 genome assembly were extracted using the MISA tool [17]. The parameters set for SSR identification included a minimum of 10 repeats for mononucleotide motifs, 6 repeats for dinucleotide motifs, and 5 repeats for tri-, tetra-, penta-, and hexanucleotide motifs, with a maximum length of compound SSRs set to 100 nucleotides and a minimum distance of 50 nucleotides between SSRs. Based on the MISA results, primer pairs were designed using Primer3 software [18], with default parameters, targeting the flanking sequences of the SSRs. To identify polymorphic SSRs and their genomic distribution, SSR mining was conducted across all three genome assemblies using the MISA tool, along with corresponding primer prediction. SSRs extracted from each genotype were compared to those from the reference genome to assess polymorphism. Both monomorphic and polymorphic SSRs were extracted, considering identical 5′ and 3′ flanking sequences of 20 nucleotides and variable numbers of SSR motifs, using Perl scripts. Hypervariable polymorphic SSR markers, defined as having a repeat length of ≥20 nucleotides, were also identified [19].

### Prediction and acquisition of noncoding RNA

The three main classes of ncRNAs based on their regulatory potential were predicted. For the identification of microRNAs (miRNAs), initially, the known miRNAs and pre-miRNAs of bitter gourd from miRBase [20] were retrieved. The duplicates were removed using CD-HIT [21]. The pre-miRNA sequences of nonredundant bitter gourd miRNAs were then aligned with the bitter gourd RefSeq genome (GCA_013281855.1) using BLASTn [22]. Sequences with zero gaps and ≤3 mismatches were selected, including 500 nucleotides of upstream and downstream flanking regions, resulting in sequences longer than 1000 nucleotides [23]. These sequences were fragmented into 200-nucleotide segments using a 25-nucleotide sliding window with SeqKit [24]. The resulting fragments were clustered again with CD-HIT to obtain nonredundant sequences. Further, nonredundant sequences were analysed for secondary structure prediction using RNAfold [25], with a minimum free energy threshold of >−20 [26]. Sequences shorter than 60 nucleotides, containing non-AUGC bases, exhibiting multiloop structures, or identified as pseudo-pre-miRNAs were excluded using the TripletSVM classifier [27]. The remaining putative pre-miRNAs were utilized for the prediction of mature miRNAs using the mature Bayes tool [28]. Finally, potential mRNA targets of the predicted miRNAs were identified using psRANTarget [29] with an expectation value threshold of 2. To identify circular RNAs (circRNAs), high-quality clean reads obtained after preprocessing were used. These reads were aligned to the bitter gourd reference genome using BWA (version 0.7.17), with the mem-T 20 option. The alignment was performed using the circRNA identification tool CIRI2 (version 2.1.1) [30]. The resulting SAM file was then analysed with the CIRI2 core programme to identify putative circRNAs.

For the identification of long noncoding RNAs (lncRNAs) from RNA-seq data across six tissues, we initially mapped the reads using HISAT2 [31] and performed assembly with StringTie (version 1.3.5) [32]. Putative lncRNAs were then predicted from the assembled reads using Coding Potential Calculator 2 (CPC2) [33] and subjected to further validation to confirm their status as noncoding transcripts. The validation steps included the following: (i) *Transcript selection criteria*: We selected transcripts based on the following criteria: length ≥200 bp, open reading frame ≤100 amino acids, strand information (± strand), and CPC2 score <0.5. These transcripts were further processed using OrfPredictor [34] and annotated with the GCF_001995035.1 genome assembly using GffCompare [35]. (ii) *Database searches*: The annotated transcripts were searched against the NCBI-nr protein database using BLASTx (*E*-value ≤0.01, coverage >80%, and identity >90%) and against the Pfam protein database using HMMER [36] to confirm noncoding status. (iii) *Expression filtering*: Transcripts were classified as lncRNAs if they met the following expression thresholds: FPKM (fragments per kilobase of transcript per million mapped reads) ≥ 0.5 for multi-exon transcripts and FPKM ≥ 1 for single-exon transcripts.

### Prediction of transcription factors and transcripts

A total of 1807 putative transcription factors were identified in the bitter gourd genome through alignment with the iTAK database [37]. The annotation files, i.e. GTF files for reads from each tissue, were consolidated into a single GTF file per tissue using the merge option in StringTie. The HISAT2-build command from HISAT2 (version 2.2.0) [31, 38] was then employed to index the reference genome with splice site and exon annotations. SAM files, produced from read alignment, were converted into binary BAM files using Samtools (version 1.9) [39]. Transcriptome assembly for each BAM file was then carried out using StringTie (version 2.1.4).

### Manual curation and inclusion of QTL

Quantitative trait loci (QTL) are crucial tools in modern breeding, enhancing our understanding of the genetic basis underlying key traits and aiding in the development of improved bitter gourd varieties. We systematically reviewed the literature to identify and compile information on established QTL for bitter gourd, curating approximately 150 entries associated with significant traits. This information has been integrated into our database, with a dedicated section for easy access. Each QTL entry includes details on the traits associated with the QTL, the names of the QTL, their positions on chromosomes, and references for further investigation.

### Database and web interface implementation

The Bitter Gourd Resource Database (BgDB) is a specialized relational database designed to manage comprehensive data for bitter gourd research. It is structured with a three-tier architecture comprising the client, server, and database layers. It is composed of a presentation layer for user interaction, the application layer for data processing and logic, and the data layer for storage and management, ensuring scalability, security, and maintainability through layer separation. It utilizes Apache (version 2.4.58) for web serving, MySQL (version 8.0.32) for data management, and PHP (Hypertext Preprocessor; version 8.2.12) for server-side scripting, following updates and modifications [40, 41]. The MySQL database contains 23 tables that store detailed information on genetic variants, ncRNAs, transcription factors, RNA transcripts, and QTL. The web interface, developed using PHP, HTML (Hypertext Markup Language), CSS, and JavaScript, provides a dynamic and interactive user experience. When a user performs a search, a PHP script processes the request, constructs an SQL (Structured Query Language) query, and retrieves the relevant data from the MySQL database. The server then dynamically generates an HTML page to display the results, ensuring efficient and accurate information delivery for research purposes.

### Functional establishment of BgDB

Genomic DNA was isolated from young leaves using a modified CTAB (cetyl trimethyl ammonium bromide) method [42]. The quality and quantity of the DNA were assessed using gel electrophoresis and a NanoDrop 8000 spectrophotometer. The isolated DNA was diluted to 50 ng/μl and stored at 4°C for subsequent use. DNA quality and quantity were further verified by running the samples on a 0.8% (w/v) agarose gel. PCR amplification was performed in a 10 μl reaction mixture containing 200 μM dNTPs, 20–25 ng of DNA, 1 μl of 10 μM primers, 1 U/μl Taq polymerase, and 1× DreamTaq buffer with 20 mM MgCl_2_. The PCR was executed using a thermal cycler with the following programme: initial denaturation at 95°C for 5 min, followed by 35 cycles of denaturation at 95°C for 1 min, annealing at 55–60°C for 45 s, and extension at 72°C for 1 min. A final extension at 72°C for 5 min was followed by a hold at 4°C. The PCR products were resolved on a 4% agarose gel stained with ethidium bromide (0.5 mg/ml). The resolution of the amplified products was assessed using a 50-bp DNA ladder (G-Bioscience). Gels were visualized using a gel documentation system (Alpha Innotech, San Leandro, CA, USA).

## Results

### Web interface and usage

**Figure 2.**
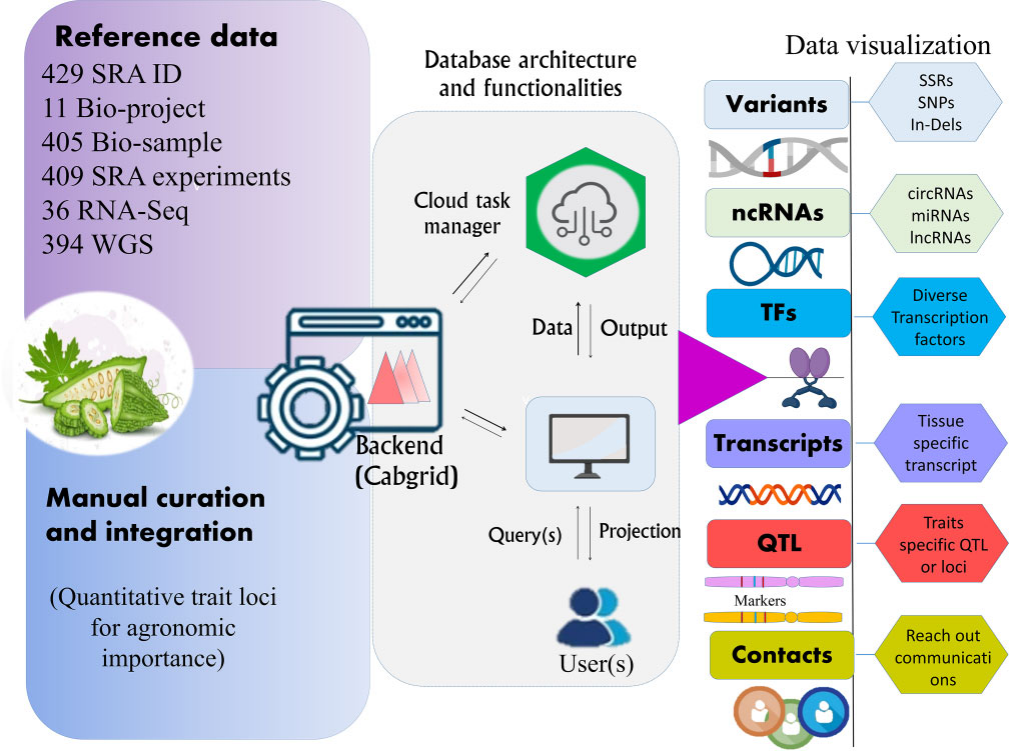
An overview of the architecture and functionality of the developed web resource for bitter gourd, designed to provide access to key molecular footprints derived from complex genomic data, supporting breeding efforts for trait improvement.

The developed BgDB offers comprehensive resource related to bitter gourd, encompassing four primary datasets. These datasets provide detailed information on the attributes, functions, descriptions, and functional footprints of bitter gourd (Fig. 2). The platform features an intuitive web interface that allows users to efficiently browse, search, and retrieve data on various molecular functional footprints associated with bitter gourd (see Supplementary Fig. S1). The top navigation bar includes six functional header menus: ‘Home’, ‘About’, ‘Search’, ‘QTL’, ‘Manual’, and ‘Contact’. These menus are designed to facilitate streamlined access to database sections. The ‘Home’ page delivers a succinct overview of the database and its core functionalities, along with essential background information on bitter gourd and the rationale behind the study. Data can be extracted, downloaded, and copied through options available within each dataset’s dashboard. Additionally, a reset option, located on the right side of the search menu, allows users to return to the initial search criteria. An independent search feature, positioned in the top-right corner, facilitates random queries for each data type, enabling users to conduct specific searches and obtain targeted results.

### Molecular footprints accession and their retrieval for downstream utility

#### Molecular variant

The icon search feature allows users to access molecular variant data by opening a window where the desired data type can be selected. There are four data types available: variants, ncRNAs, transcription factors, and transcripts. Upon selecting ‘variants’, a new window will prompt the user to choose the specific type of variant. The available variant types include SSRs , SNP, and Indels. After selecting a variant type, the system will prompt the user to choose a chromosome. A detailed table will then be generated, displaying information on the chosen chromosome, including motif repeat types, start location, length, forward and reverse primer details, melting temperature, amplicon size, and types of polymorphism. For example, chromosome 1 has 14 799 identified SSRs. Users can similarly access polymorphisms and monomorphic SSR markers across chromosomes by selecting them individually. For SNPs, after selecting ‘SNP’ instead of ‘SSR’, a new window will open allowing the user to choose from four different bioProjects publicly available for bitter gourd. Clicking on a required bioProject will open another window to select the chromosome. For instance, selecting chromosome 1 will display detailed information including variant location, nucleotide changes relative to the reference genome, and quality scores. This information is available for all SNPs across chromosomes. For Indels, selecting ‘Indel’ from the variant options will provide comprehensive information on all Indels present across the chromosomes.

### Regulatory ncRNAs

A search option is facilitated for another significant class of RNA known as regulatory RNA. By selecting the ‘noncoding RNA’ data type from the search icon, users are directed to a new window where they can choose from three main classes of ncRNAs: circRNAs, miRNAs, and lncRNAs. Upon selecting a specific class of ncRNA, the system will prompt users to choose a tissue type. For example, selecting ‘fruits’ will provide details on circRNAs expressed in fruit tissue of bitter gourd. Similar information is available for other tissue types, including leaf, ovary, root, stem, and seed. Users can also retrieve information for all tissues simultaneously by selecting the ‘all’ option, which includes 2426 entries. For miRNAs, selecting this class from the ncRNA tab provides a chromosome-wise alignment of mature miRNA sequences, including start-to-end alignment, prediction of precursor sequences, duplex positions, and sequences. Apart from their chromosomal origin, a repertoire of miRNAs is represented by their position on the scaffold, indicating their origin in genome assemblies, and can often be used to guide their target mRNAs. It also includes the positions and sequences of mature miRNAs. A total of 806 mature reads were partially or fully aligned with mature miRNAs from other species, suggesting their potential prevalence. When selecting ‘lncRNA’ from the RNA type icon, users will access information on 4817 putative lncRNA sequences. This information includes their prevalence in different tissues and chromosomes, start and end positions, and lengths identified through contemporary reference genes and genomes. Additionally, the data provide class codes, exon numbers, covariance, FPKM values, and involved pathways, offering critical insights for user evaluation and downstream analysis.

### Transcription factors and regulators

Chromosome-wise predictions of transcription factors, including their relative locations on the chromosome with start and end positions, are displayed when selecting ‘transcription factor’ from the search tab, followed by choosing the data type. These predictions are categorized into two broad domains: transcription factors and transcription regulators. The database includes a total of 1220 transcription factors with 50 distinct subtypes, and 295 transcription regulators with 24 subtypes, facilitating detailed visualization and effective utilization for downstream research. The transcription factors and regulators can be further explored based on their specific locations on the chromosome.

### Transcript signature

In the search tab, one of the most significant biological entities, i.e. *transcripts*, is included. By selecting ‘transcript’ from the data type tab, users will access a new window with two sub-tabs: one for searching based on known gene IDs and another for exploring expression status and other relevant information specific to selected tissues. Three distinct icons are available in the transcript search section, allowing users to choose, based on their specific needs and preferences. Users can obtain relative expression values in FPKM for various tissues and chromosomes. The data include gene length, reference gene length, coding probability, number of exons, and coverage in relation to FPKM values. By selecting different tissues, users can view FPKM values and coverage details, providing insights into the expression status of genes of interest.

### QTL: manual annotation and viewer

QTL are essential tools in breeding programmes. Therefore, manual curation has been done and a comprehensive set of validated QTL from published literature on bitter gourd has been included. Users can view detailed information on significant traits, including the names of the identified underlying QTL, their relative positions on chromosomes, and relevant references for further study. Search for QTL by trait or chromosome, using the search options provided in the QTL page menu, can be made. For example, if a user wants to find QTL related to the trait ‘bitterness’, selecting this trait from the navigation bar will directly display the relevant results. Similarly, users can choose to view QTL based on specific chromosomes by selecting the desired chromosome from the navigation menu. This visualization of QTL in the database facilitates the identification of relevant QTL information for specific traits, which can be valuable for enhancing breeding initiatives.

### Utility of the BgDB database

**Figure 3.**
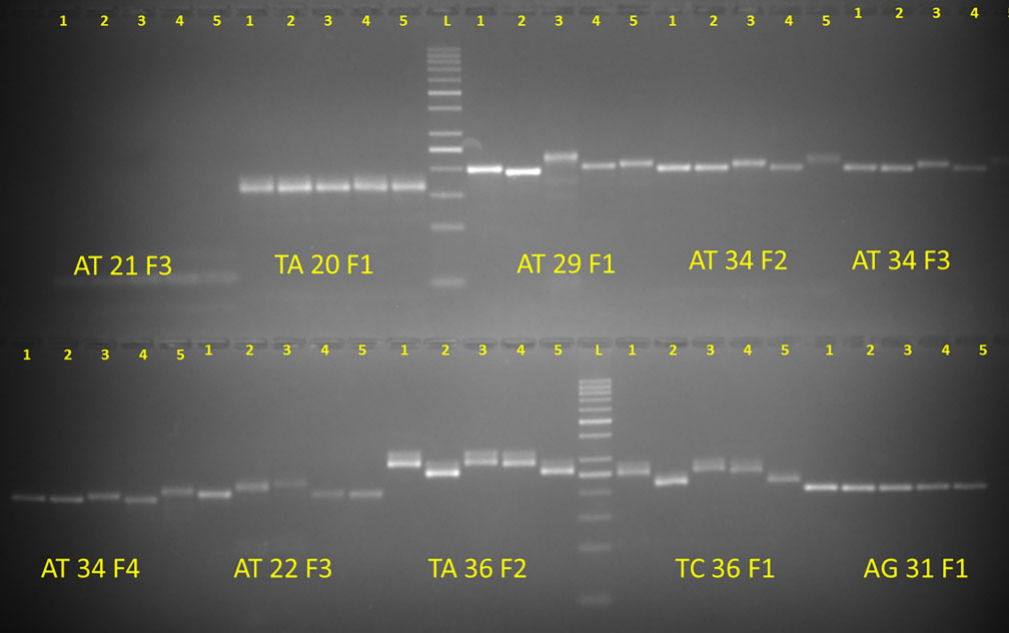
Wet-lab validation of a set of molecular markers from the developed genomic resource.

The development of a database achieves significance when it supports functional scientific applications. To validate its utility, a random pool of SSR markers across five diverse bitter gourd genotypes were tested. A total of 51 SSR primers (with ≤20 tentative primers) and 50 primers (with ≤8 tentative primers), which were uniformly distributed throughout the genome, for validation in five commercially cultivated bitter gourd genotypes (Pusa Mousomi, Pusa Vishesh, Pusa Rasdhar, Pusa Aushadhi, and Pusa Poorvi) were selected. Among the 51 SSR primers, 48 (94.1%) produced clear banding patterns, while 3 markers failed to amplify. Out of these 51 SSRs, 35 (68.6%) displayed polymorphisms among the five genotypes. For the 50 primers in the second set, 32 (64.0%) yielded clear banding patterns, and 17 (34%) of these were polymorphic, successfully differentiating the five bitter gourd genotypes (Fig. 3 and Supplementary Table S1).

## Discussion

The bitter gourd database, BgDB, is a valuable resource for bitter gourd breeders and researchers. Its customization options for chromosome-based searches facilitate the mapping of molecular variants, ncRNAs, transcription factors, and transcripts, which are crucial for the improvement of bitter gourd. This tool can be utilized by biologists involved in diverse research objectives related to bitter gourd, including the development of molecular variants and the investigation of the functional significance of these markers in gene regulation and genome evolution for agronomically advantageous traits [12, 43].

The development of user-friendly and PCR-based molecular markers is instrumental in genomics-related studies on crops like bitter gourd, which have limited genomic resources [44]. Omics-based research in plants has been revolutionized with the application of next-generation sequencing (NGS) technologies, coupled with computational methods [45]. NGS-based information is instrumental in designing and developing highly efficient SSR markers for their use in future studies. To undertake molecular genetics and genomic studies, SSR markers have proven to be very useful, in addition to their applications in evolutionary and phylogenetic studies [46]. Precise amplification of >90% of the genic SSRs and polymorphic amplification patterns in around 30% of the SSRs indicated the usefulness of the developed microsatellite-based markers in future molecular genetics studies in important nutritionally dense vegetable crops like bitter gourd. This case study established the reliability of the developed genomic resources and their use in trait discovery and functional genomics in bitter gourd.

The predesigned primers for PCR amplification of specific motifs available in the database can aid studies on mutability, microsatellite abundance, and their associations with particular diseases or phenotypes. This functionality may also support cross-species transferability studies [47]. The database provides detailed information on chromosome-specific motif repeats, including their start locations, lengths, forward and reverse primer details, melting temperatures, amplicon sizes, and types of polymorphisms [43, 47]. Additionally, users can explore important classes of tissue-specific RNA, including miRNAs [48], circRNAs, and lncRNAs. The database visualizes their chromosome-wise alignment, precursor sequences, duplex positions, and mature sequences. Prevalence data for these RNAs in different tissues and chromosomes, along with their start and end positions and lengths, are identified through contemporary gene and genome annotations [48, 49].

The database also visualizes predicted transcription factors and their relative chromosome locations, including start and end positions. This feature is valuable for downstream applications in research [50, 51]. Furthermore, transcript signatures, which are critical biological entities, include expressive elements and provide relative expression values in FPKM across tissues and chromosomes. Information on gene length, reference gene length, coding probability, exon numbers, and coverage relative to FPKM values is available and essential for hypothesis generation and result interpretation [12, 50, 51].

QTL are crucial tools for breeding programmes, and our developed database includes comprehensively validated QTL from the published literature on bitter gourd. Users can view significant traits, identified QTL names, their relative chromosome positions, and references for further details [52]. The database’s significance is underscored by its practical application in scientific research. For example, we validated SSR markers by amplifying and displaying clear banding patterns, which successfully differentiated the five bitter gourd genotypes from a random pool of SSR markers [12, 42, 43, 47]. Overall, the database holds significant potential to advance research in the genetic improvement, cultivation, and utilization of bitter gourd, thereby enhancing agricultural practices and contributing to scientific knowledge within the field. Furthermore, this user-friendly web resource offers easy access to and retrieval of molecular and omics data, facilitating the development of novel bitter gourd genotypes through molecular breeding and biotechnological interventions.

## Conclusion

Bitter gourd, a medicinal vegetable renowned for its numerous health benefits, requires ongoing breeding efforts to meet consumer demands. However, its molecular characteristics are less well studied compared to other cash crops, and the available information is sparse and fragmented, posing a significant challenge for scientific research and practical applications. To address this issue, a user-friendly, three-tier architecture database, BgDB, is developed that offers comprehensive information on regulatory transcripts, differentially expressed transcripts, molecular variants and their associated flanking primers, transcription factors, QTL, and regulatory ncRNAs. This database provides extensive search options for both simple and compound molecular variants, as well as other related features, enabling users to explore new research avenues in bitter gourd. The selected microsatellite markers were validated for differentiating bitter gourd genotypes, ensuring the precise utility and reproducibility of the designed web resources. These resources can be employed for genetic diversity analysis, varietal differentiation, trait fine-tuning, biotechnological interventions, and the discovery of QTL for improving bitter gourd varieties.

## Supplementary Material

baaf039_Supplemental_Files

## Data Availability

The data is freely accessible online through BgDB at https://bgdb.daasbioinfromaticsteam.in/index.php.

## References

[1] Gayathry KS, John JA. A comprehensive review on bitter gourd (*Momordica charantia* L.) as a gold mine of functional bioactive components for therapeutic foods. Food Prod Process Nutr. 2022;4:10. 10.1186/s43014-022-00089-x.

[2] Wang H, Shuai X, Ye S et al. Recent advances in the development of bitter gourd seed oil: from chemical composition to potential applications. Crit Rev Food Sci Nutr. 2023;63:10678–90. 10.1080/10408398.2022.2081961. 35648048

[3] Yoshime LT, de Melo ILP, Sattler JAG et al. Bitter gourd (*Momordica charantia* L.) seed oil as a naturally rich source of bioactive compounds for nutraceutical purposes. Nutrire. 2016;41:12. 10.1186/s41110-016-0013-y.

[4] Farooqi AA, Khalid S, Tahir F et al. Bitter gourd (*Momordica charantia*) as a rich source of bioactive components to combat cancer naturally: are we on the right track to fully unlock its potential as inhibitor of deregulated signaling pathways. Food Chem Toxicol. 2018;119:98–105. 10.1016/j.fct.2018.05.024. 29753870

[5] Sun L, Zhang X, Dong L et al. The triterpenoids of the bitter gourd (*Momordica charantia*) and their pharmacological activities: a review. J Food Compos Anal. 2021;96:103726. 10.1016/j.jfca.2020.103726.

[6] Tan SP, Kha TC, Parks SE et al. Bitter melon (*Momordica charantia* L.) bioactive composition and health benefits: a review. Food Rev Int. 2016;32:181–202. 10.1080/87559129.2015.1057843.

[7] Banerjee J, Chanda R, Samadder A. Anti-diabetic activity of *Momordica charantia* or bitter melon: a review. Acta Sci Pharm Sci. 2019;3:24–30.

[8] Batool F, Adeel S, Iqbal N et al. Sustainable natural coloring potential of bitter gourd (*Momordica charantia* L.) residues for cotton dyeing: innovative approach towards textile industry. Environ Sci Pollut Res. 2022;29:34974–83. 10.1007/s11356-021-17803-w. 35040061

[9] Akhtar S, Husain Q. Potential applications of immobilized bitter gourd (*Momordica charantia*) peroxidase in the removal of phenols from polluted water. Chemosphere. 2006;65:1228–35. 10.1016/j.chemosphere.2006.04.049. 16764905

[10] Upadhyay A, Agrahari P, Singh DK. A review on salient pharmacological features of *Momordica charantia*. Int J Pharmacol. 2015;11:405–13. 10.3923/ijp.2015.405.413.

[11] Matsumura H, Hsiao MC, Lin YP et al. Long-read bitter gourd (*Momordica charantia*) genome and the genomic architecture of nonclassic domestication. Proc Natl Acad Sci USA. 2020;117:14543–51. 10.1073/pnas.1921016117. 32461376 PMC7321978

[12] Baluchamy N, Thayyil P, Mathew D et al. Candidate gene based SSR and SNP markers for gynoecy in bitter gourd (*Momordica charantia* L.). Mol Biol Rep. 2023;50:1125–32. 10.1007/s11033-022-08098-2. 36401706

[13] Andrews S . FastQC—A Quality Control Tool for High Throughput Sequence Data. Babraham Bioinformatics, 2010.

[14] Prjibelski A, Antipov D, Meleshko D et al. Using SPAdes De Novo Assembler. Curr Protoc Bioinform. 2020;70:e102. 10.1002/cpbi.102. 32559359

[15] Bolger AM, Lohse M, Usadel B. Trimmomatic: a flexible trimmer for Illumina sequence data. Bioinformatics. 2014;30:2114–20. 10.1093/bioinformatics/btu170. 24695404 PMC4103590

[16] Langmead B, Salzberg SL. Fast gapped-read alignment with Bowtie 2. Nat Methods. 2012;9:357–59. 10.1038/nmeth.1923. 22388286 PMC3322381

[17] Beier S, Thiel T, Münch T et al. MISA-web: a web server for microsatellite prediction. Bioinformatics. 2017;33:2583–85. 10.1093/bioinformatics/btx198. 28398459 PMC5870701

[18] Untergasser A, Cutcutache I, Koressaar T et al. Primer3—new capabilities and interfaces. Nucleic Acids Res. 2012;40:e115. 10.1093/nar/gks596. 22730293 PMC3424584

[19] Temnykh S, DeClerck G, Lukashova A et al. Computational and experimental analysis of microsatellites in rice (*Oryza sativa* L.): frequency, length variation, transposon associations, and genetic marker potential. Genome Res. 2001;11:1441–52. 10.1101/gr.184001. 11483586 PMC311097

[20] Griffiths-Jones S . miRBase: microRNA sequences, targets and gene nomenclature. Nucleic Acids Res. 2006;34:D140–4. 10.1093/nar/gkj112. 16381832 PMC1347474

[21] Huang Y, Niu B, Gao Y et al. CD-HIT suite: a web server for clustering and comparing biological sequences. Bioinformatics. 2010;26:680–82. 10.1093/bioinformatics/btq003. 20053844 PMC2828112

[22] Camacho C, Coulouris G, Avagyan V et al. BLAST+: architecture and applications. BMC Bioinformatics. 2009;10:421. 10.1186/1471-2105-10-421. 20003500 PMC2803857

[23] Altschul SF . Basic local alignment search tool. J Mol Biol. 1990;215:403–10. 10.1016/S0022-2836(05)80360-2. 2231712

[24] Shen W, Le S, Li Y et al. SeqKit: a cross-platform and ultrafast toolkit for FASTA/Q file manipulation. PLoS One. 2016;11:e0163962. 10.1371/journal.pone.0163962. 27706213 PMC5051824

[25] Lorenz R, Luntzer D, Hofacker IL et al. SHAPE directed RNA folding. Bioinformatics. 2016;32:145–47. 10.1093/bioinformatics/btv523. 26353838 PMC4681990

[26] Lorenz WA, Clote P. Computing the partition function for kinetically trapped RNA secondary structures. PLoS One. 2011;6:e16178. 10.1371/journal.pone.0016178. 21297972 PMC3030561

[27] Xue C, Li F, He T et al. Classification of real and pseudo microRNA precursors using local structure-sequence features and support vector machine. BMC Bioinformatics. 2005;6:310. 10.1186/1471-2105-6-310. 16381612 PMC1360673

[28] Leclercq M, Diallo AB, Blanchette M. Computational prediction of the localization of microRNAs within their pre-miRNA. Nucleic Acids Res. 2013;41:7200–11. 10.1093/nar/gkt466. 23748953 PMC3753617

[29] Dai X, Zhao PX. psRNATarget: a plant small RNA target analysis server. Nucleic Acids Res. 2011;39:W155–9. 10.1093/nar/gkr319. 21622958 PMC3125753

[30] Gao Y, Zhang J, Zhao F. Circular RNA identification based on multiple seed matching. Brief Bioinform. 2018;19:803–10. 10.1093/bib/bbx014. 28334140

[31] Kim D, Paggi JM, Park C et al. Graph-based genome alignment and genotyping with HISAT2 and HISAT-genotype. Nat Biotechnol. 2019;37:907–15. 10.1038/s41587-019-0201-4. 31375807 PMC7605509

[32] Pertea M, Pertea GM, Antonescu CM et al. StringTie enables improved reconstruction of a transcriptome from RNA-seq reads. Nat Biotechnol. 2015;33:290–95. 10.1038/nbt.3122. 25690850 PMC4643835

[33] Kang YJ, Yang DC, Kong L et al. CPC2: a fast and accurate coding potential calculator based on sequence intrinsic features. Nucleic Acids Res. 2017;45:W12–6. 10.1093/nar/gkx428. 28521017 PMC5793834

[34] Min XJ, Butler G, Storms R et al. OrfPredictor: predicting protein-coding regions in EST-derived sequences. Nucleic Acids Res. 2005;33:W677–80. 10.1093/nar/gki394. 15980561 PMC1160155

[35] Pertea G, Pertea M. GFF utilities: gffRead and GffCompare. F1000Research. 2020;9:304. 10.12688/f1000research.23297.1. PMC7222033 32489650

[36] Finn RD, Clements J, Eddy SR. HMMER web server: interactive sequence similarity searching. Nucleic Acids Res. 2011;39:W29–37. 10.1093/nar/gkr367. 21593126 PMC3125773

[37] Zheng Y, Jiao C, Sun H et al. iTAK: a program for genome-wide prediction and classification of plant transcription factors, transcriptional regulators, and protein kinases. Mol Plant. 2016;9:1667–70. 10.1016/j.molp.2016.09.014. 27717919

[38] Pertea M, Kim D, Pertea GM et al. Transcript-level expression analysis of RNA-seq experiments with HISAT, StringTie and Ballgown. Nat Protoc. 2016;11:1650–67. 10.1038/nprot.2016.095. 27560171 PMC5032908

[39] Li H, Handsaker B, Wysoker A et al. The Sequence Alignment/Map format and SAMtools. Bioinformatics. 2009;25:2078–79. 10.1093/bioinformatics/btp352. 19505943 PMC2723002

[40] Sarika J, Arora Vasu, Iquebal MA et al. PIPEMicroDB: microsatellite database and primer generation tool for pigeonpea genome. Database. 2013;2013:bas054. 10.1093/database/bas054. 23396298 PMC3567484

[41] Jasrotia RS, Yadav PK, Iquebal MA et al. VigSatDB: genome-wide microsatellite DNA marker database of three species of *Vigna* for germplasm characterization and improvement. Database. 2019;2019:baz055. 10.1093/database/baz055 31147679 PMC6542692

[42] Rathod V, Behera TK, Gaikwad AB et al. Genetic analysis and tagging of gene controlling fruit tubercles and fruit ridgeness pattern in bitter gourd using SSR markers. Indian J Genet Plant Breed. 2019;79:749–755. 10.31742/IJGPB.79.4.14

[43] Behera TK, Singh AK, Staub JE. Comparative analysis of genetic diversity in Indian bitter gourd (*Momordica charantia* L.) using RAPD and ISSR markers for developing crop improvement strategies. Sci Hortic. 2008;115:209–17. 10.1016/j.scienta.2007.08.013.

[44] Kuang Z, Xiao C, Ilyas MK et al. Use of SSR markers for the exploration of genetic diversity and DNA finger-printing in early-maturing upland cotton (*Gossypium hirsutum* L.) for future breeding program. Agronomy. 2022;12:1513. 10.3390/agronomy12071513.

[45] Yadav P, Vaidya E, Rani R et al. Recent perspective of next generation sequencing: applications in molecular plant biology and crop improvement. Proc Natl Acad Sci India Sect B: Biol Sci. 2018;88:435–49. 10.1007/s40011-016-0770-7.

[46] Varshney RK, Graner A, Sorrells ME. Genic microsatellite markers in plants: features and applications. Trends Biotechnol. 2005;23:48–55. 10.1016/j.tibtech.2004.11.005. 15629858

[47] Saxena S, Singh A, Archak S et al. Development of novel simple sequence repeat markers in bitter gourd (*Momordica charantia* L.) through enriched genomic libraries and their utilization in analysis of genetic diversity and cross-species transferability. Appl Biochem Biotechnol. 2015;175:93–118. 10.1007/s12010-014-1249-8. 25240849

[48] Thirugnanasambantham K, Saravanan S, Karikalan K et al. Identification of evolutionarily conserved *Momordica charantia* microRNAs using computational approach and its utility in phylogeny analysis. Comput Biol Chem. 2015;58:25–39. 10.1016/j.compbiolchem.2015.04.011. 25988220

[49] Villalba-Bermell P, Marquez-Molins J, Gomez G. Identification, characterization and transcriptional analysis of the long non-coding RNA landscape in the family *Cucurbitaceae*. bioRxiv, 10.1101/2024.01.12.575433, 15 January 2024, preprint: not peer reviewed.

[50] Li Y, Wu J, Fan Y et al. Evaluation of morphological traits, hormonal metabolism, and transcriptional abundance in bitter gourd (*Momordica charantia* L.) plants in response to ethephon inducement. Sci Hortic. 2021;282:110033. 10.1016/j.scienta.2021.110033.

[51] Ning Y, Liu Z, Liu J et al. Comparative transcriptomics analysis of tolerant and sensitive genotypes reveals genes involved in the response to cold stress in bitter gourd (*Momordica charantia* L.). Sci Rep. 2024;14:16564. 10.1038/s41598-024-58754-9. 39019887 PMC11255239

[52] Rao PG . Recent advances in breeding of bitter gourd (*Momordica charantia* L.). In: Al-Khayri, Jain, Johnson (eds.), Advances in Plant Breeding Strategies: Vegetable Crops. Cham: Springer International Publishing, 2021, p. 87–121. 10.1007/978-3-030-66961-4

