# Supplementary material for: BgDB: a comprehensive genomic resource information system of bitter gourd for accelerated breeding programme
Source: Database (Oxford). 2025 Sep 24;2025:baaf039. doi: 10.1093/database/baaf039 (PMC12462627; doi:10.1093/database/baaf039)
Supplement: baaf039_Supplemental_Files [file baaf039_supplemental_files.zip › Supplementary table.docx]

Supplementary table 1. Co-dominant markers genotyping on Bitter gourd lines

| **Marker** | **PDM** | **Pusa Vishesh** | **Pusa Rasdhar** | **Pusa Aushadhi** | **Pusa Poorvi** |
| --- | --- | --- | --- | --- | --- |
| AT 24 | 120 | 120 | 140 | 120 | 120 |
| AT 22F1 | 150/100 | 150/100 | 150/100 | 150/100 | 150/100 |
| AT 22 F3 | 200 | 200 | 250/200 | 200 |  |
| AT 34 F1 | 180 | 180 | 200 | 180 | 200 |
| AG 58 F1 | 150 | 170 | 170 | 150 | 150 |
| AG 58F2 | 150 | 170 | 170 | 150 | 150 |
| AG 43 F1 | 220 | 220 | 240 | 220 | 220 |
| AG 43F2 | 220 | 220 | 240 | 220 | 220 |
| AT 21 F3 | 270 | 270 | 270 | 270 | 270 |
| AT 21 F4 | 250 | 250 | 260 | 250 | 250 |
| AT 21 F5 | 200 | 200 | 200 | 200 | 200 |
| AT 25 F1 | 120 | 120 | 120 | 120 | 120 |
| GA 22 F1 | 170 | 170 | 150 | 170 | 150 |
| TA 20 F1 | 190 | 190 | 220 | 190 | 190 |
| AT 21 F4 | NA | NA | NA | NA | NA |
| GA 22 F2 | 220 | 220 | 200 | 220 | 200 |
| GA22 F3 | 220 | 220 | 200 | 220 | 220 |
| AT22 F1 | 200 | 200 | 250 | 200 |  |
| TA 20 F1 | 170 | 170 | 170 | 170 | 170 |
| AT 29 F1 | 200 | 180 | 240 | 180 | 200 |
| AT 34 F2 | 180 | 180 | 200 | 180 | 200 |
| AT 34 F3 | 180 | 180 | 200 | 180 | 200 |
| AT 34 F4 | 180 | 180 | 200 | 180 | 210 |
| AT 22 F3 | 200 | 230 | 240 | 200 | 200 |
| TA 36 F2 | 350/300 | 300/250 | 350/300 | 350/300 | 300/250 |
| TC 36 F1 | 270/200 | 220 | 270/200 | 270/200 | 220 |
| AG 31 F1 | 200 | 200 | 200 | 200 | 200 |
| AG 31 F2 | 200 | 200 | 200 | 200 | 200 |
| TA 20 F2 | 240/220 | 240/220 | 240/220 | 240/220 | 240/220 |
| TA 26 F2 | 310/270 | 310/270 | 310/270 | 310/270 | 310/270 |
| TA 26 F3 | 310/270 | 310/270 | 310/270 | 310/270 | 310/270 |
| TA 20 F2 | 200 | 200 | 250 | 200 | 200 |
| TA 20 F3 | 270/250 | 270/250 | 270/250 | 270/250 | 270/250 |
| AT 21 F | 250/230 | 250/230 | 250/230 | 250/230 | 250/230 |
| AT 26 F1 | 310/250 | 310/250 | 310/250 | 310/250 | 310/250 |
| AT 21 F2 | 200 | 200 | 200 | 200 | 200 |
| AT 21 F5 | 270 | 270 | 300 | 270 | 270 |
| AT 21 F2 | 250 | 250 | 270 | 250 | 270 |
| AT 22 F1 | 200 | 230 | 240 | 200 | 200 |
| AT 21 F | 170 | 170 | 170 | 170 | 170 |
| AT 23 F1 | 260/250/200 | 260/250/200 | 260/250/200 | 260/250/200 | 260/250/200 |
| AT 23 F2 | 190/140 | 190/140 | 190/140 | 190/140 | 190/140 |
| AT 23 F3 | 250/200 | 250/200 | 250/200 | 250/200 | 250/200 |
| TA 26 F1 | 260/250 | 260/250 | 260/250 | 260/250 | 260/250 |
| 1AT 22F2 | 200 | 200 | 200 | 230 | 230 |
| 2AT22F2 | 200 | 200 | 230 | 200 | 230 |
| 3AT 22F2 | 200 | 230 | 240/230 | 200 | 200 |
| 1AT 21F1 | 180 | 180 | 180 | 180 | 180 |
| 2AT 21 F1 | 250 | 250 | 270 | 250 | 250 |
| (AG)31 | 200 | 220 | 220 | 200 | 200 |
| (AT)26 | 150 | 150 | 200 | 150 | 150 |
| (AT)26 | 140 | 140 | 140 | 140 | 140 |
| (AT)26 | 150 | 170 | 170 | 150 | 150 |
| (AT)34 | 260 | 260/280 | 280 | 260 | 260 |
| (CT)20 | 270 | 270 | 270 | 270 | 270 |
| (CTT)23 | 300 | 250 | 300 | 300 | 250 |
| (CTT)23 | 310/270 | 310/270 | 310/270 | 310/270 | 310/270 |
| (CTT)23 | 310/250 | 310/250 | 310/250 | 310/250 | 310/250 |
| (CTT)23 | 350/300 | 300/250 | 350/300 | 350/300 | 300/250 |
| (GA)20 | 120 | 120 | 140 | 120 | 120 |
| (GA)20 | 130 | 110 | 110 | 130 | 130 |
| (GA)20 | 140 | 140 | 170 | 140 | 140 |
| (GA)20 | 150 | 150 | 150 | 150 | 150 |
| (TA)22 | 250 | 250 | 240 | 250 | 240 |
| (TA)22 | 250 | 250 | 250 | 250 | 250 |
| (TA)22 | 250 | 260 | 270 | 250 | 270 |
| (TA)22 | 250 | 250 | 250 | 250 | 250 |
| (TA)22 | 250 | 250 | 250 | 250 | 250 |
| (TA)24 | 320 | 300 | 300 | 300 | 320 |
| (TA)39 | 230 | 230 | 230 | 230 | 230 |
| (TA)39 | 200 | 200 | 230 | 200 | 230 |
| (TA)39 | 250 | 240 | 240 | 250 | 240 |
| (TA)39 | 260 | 260 | 260 | 260 | 260 |
| (TA)39 | 260 | 260 | 260 | 260 | 260 |
| (TC)20 | 200 | 230 | 200 | 200 | 230 |
| (TC)20 | 180 | 180 | 200 | 180 | 200 |
| (TC)20 | 200 | 200 | 180 | 200 | 200 |
| (TC)26 | 200 | 200 | 250 | 200 | 220 |
| (AAG)14 | 250 | 250 | 250 | 250 | 250 |
| (AAT)11 | 250 | 250 | 250 | 250 | 250 |
| (AAT)11 | 230 | 250 | 250 | 250 | 250 |
| (AAT)11 | 250 | 250 | 250 | 250 | 250 |
| (AAT)11 | 200 | 200 | 200 | 200 | 200 |
| (AAT)11 | 210 | 230 | 220 | 210 | 210 |
| (AAT)12 | 120 | 120 | 120 | 120 | 120 |
| (AAT)12 | 140 | 140 | 140 | 140 | 150 |
| (AAT)8 | 270 | 270 | 270 | 270 | 270 |
| (AAT)8 | 270 | 270 | 270 | 270 | 270 |
| (AAT)8 | 270 | 270 | 270 | 270 | 270 |
| (AAT)8 | 320 | 320 | 320 | 320 | 320 |
| (AAT)8 | 300 | 300 | 300 | 300 | 300 |
| (AAT)8 | 130 | 100 | 130 | 130 | 100 |
| (AAT)8 | 200 | 200 | 200 | 210 | 210 |
| (AAT)8 | 180 | 180 | 180 | 180 | 180 |
| (AAT)8 | 180 | 200 | 200 | 180 | 180 |
| (AAT)9 | 250/230 | 250/230 | 250/230 | 250/230 | 250/230 |
| (AAT)9 | 180/150 | 180/150 | 180/150 | 180/150 | 180/150 |
| (AAT)9 | 250 | 250 | 250 | 250 | 250 |
| (AAT)9 | 240 | 250 | 250 | 250 | 250 |
| (AAT)9 | 250 | 250 | 250 | 220 | 220 |
| (AC)14 | 270 | 270 | 270 | 270 | 270 |
