# Supplementary figures and images for: BgDB: a comprehensive genomic resource information system of bitter gourd for accelerated breeding programme
Source: Database (Oxford). 2025 Sep 24;2025:baaf039. doi: 10.1093/database/baaf039 (PMC12462627; doi:10.1093/database/baaf039)

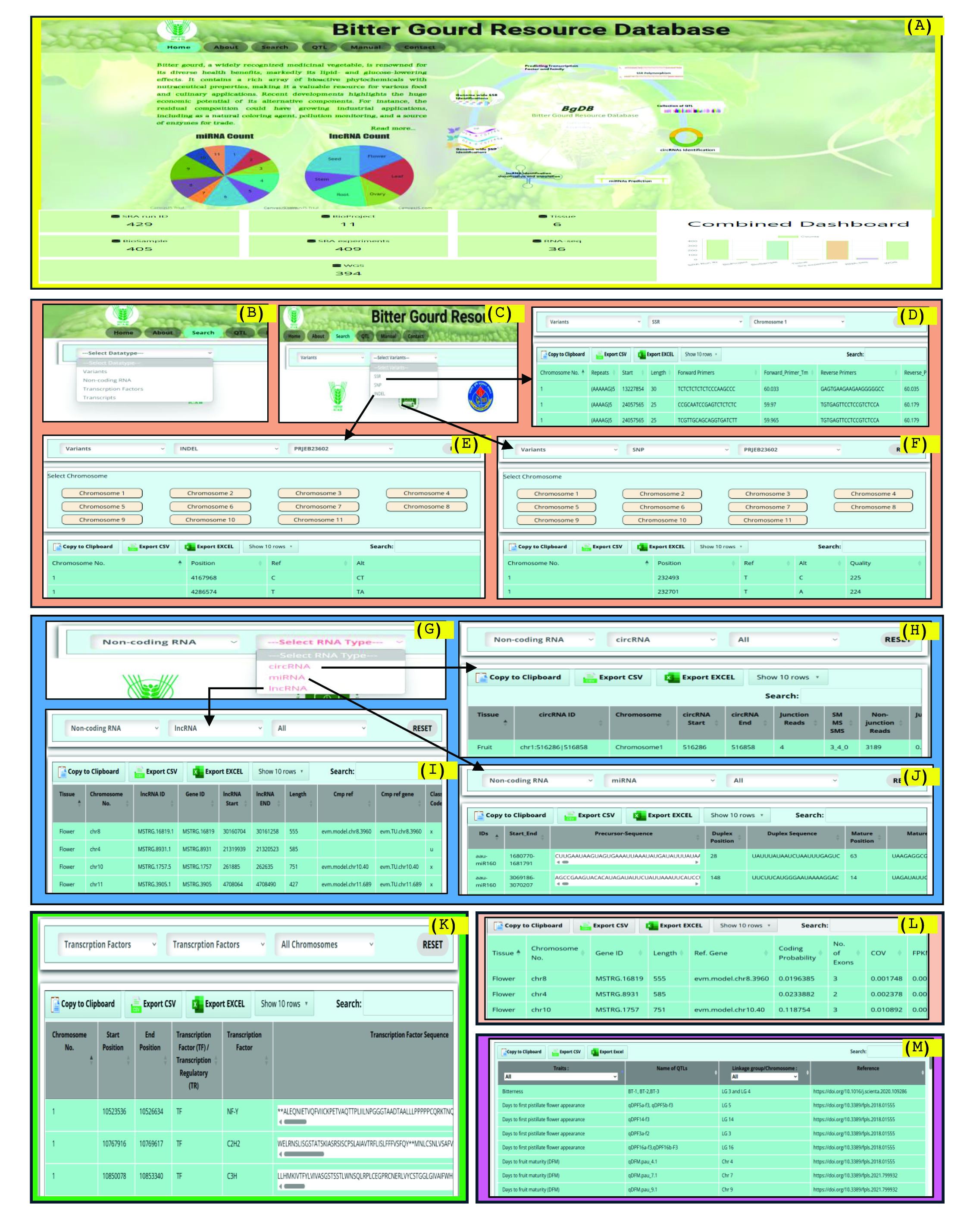

Supplement: baaf039_Supplemental_Files [file baaf039_supplemental_files.zip › Supplementary Figure 1.tif]
